# Structure Modeling and Virtual Screening with HCAR3 to Discover Potential Therapeutic Molecules

**DOI:** 10.3390/ph18091290

**Published:** 2025-08-28

**Authors:** Yulan Liu, Yunlu Peng, Zhihao Zhao, Yilin Guo, Bin Lin, Ying-Chih Chiang

**Affiliations:** 1 Kobilka Institute of Innovative Drug Discovery, School of Medicine, The Chinese University of Hong Kong—Shenzhen, Shenzhen 518172, China; 2 Key Laboratory of Structure-Based Drug Design and Discovery of Ministry of Education, Shenyang Pharmaceutical University, Shenyang 110016, China; 3 Wuya College of Innovation, Shenyang Pharmaceutical University, Shenyang 110016, China

**Keywords:** hydroxycarboxylic acid receptor 3, molecular dynamics simulations, virtual screening

## Abstract

**Background**: Hydroxycarboxylic acid receptor 3 (HCAR3) is a receptor that is mainly expressed in human adipose tissue. It can inhibit lipolysis through the inhibition of adenylyl cyclase; thus, it is closely related to the regulation of lipids in the human body. This makes HCAR3 a compelling target for developing drugs against dyslipidemia. Notably, the reported active compounds for HCAR3 are all carboxylic acids. This observation is in line with the fact that ARG111 has been reported as the key residue to anchor the active compound in a closely related homologous protein—HCAR2. **Methods**: In this study, we aim to discover new chemicals, through virtual screening, that may bind with HCAR3. As there are several choices for the receptor conformation, cross-docking was conducted and the root-mean-square deviation of the docking pose from the conformation of the crystal ligand was employed to determine the best receptor conformation for screening. Ligands from the ZINC20 database were screened through molecular docking, and 30 candidates were subjected to 100 ns MD simulations. Six stable complexes were further assessed by umbrella sampling to estimate binding affinity. **Results**: The homology model (*HCAR3_homology*) was selected as the receptor. Following the protocol determined by the retrospective docking process, prospective docking was conducted to screen the ligands from the ZINC20 database. Subsequently, the top 30 compounds with a good docking score and a good interaction with ARG111 were subjected to 100 ns molecular dynamics (MD) simulations, and their binding stability was analyzed based on the resulting trajectories. Finally, six compounds were chosen for binding free energy calculation using umbrella sampling; all showed negative binding affinities. **Conclusions:** All six compounds selected for umbrella sampling showed negative binding affinities, suggesting their potential as novel HCAR3 ligands for the development of drugs against dyslipidemia.

## 1. Introduction

Dyslipidemia, which is characterized by abnormal elevations or reductions in blood lipid levels—primarily cholesterol and triglycerides—is a major modifiable risk factor for cardiovascular diseases (CVDs), including ischemic heart disease and stroke [1,2,3,4]. Projections indicate that ischemic heart disease and stroke will be among the leading causes of disability-adjusted life years (DALYs) worldwide by 2050 [5,6]. DALYs is a widely used metric for quantifying the overall burden of disease, combining years of life lost due to premature mortality with years lived with a disability.

The findings of the 2021 Global Burden of Disease Study [5] highlight a continued global shift in disease burden from communicable to non-communicable diseases (NCDs), with metabolic risk factors, such as dyslipidemia, playing a central role in this transition [7]. In particular, targeted interventions addressing dyslipidemia and related metabolic risks could reduce the burden of DALYs by up to 23.2% in high-risk regions [5], underscoring the substantial public health value of lipid-lowering strategies. Beyond its role in CVDs, dyslipidemia is closely associated with a broad spectrum of chronic diseases, including hypertension, diabetes mellitus, and non-alcoholic fatty liver disease [8,9,10,11].

Hydroxycarboxylic acid receptor 3 (HCAR3), a rhodopsin-like G protein-coupled receptor (GPCR), belongs to a subfamily of hydroxycarboxylic acid receptors; it is known for its broad responses to chemically diverse ligands [12]. HCAR3 expression is restricted to the cell membrane in adipocytes of higher primates [13,14]. It is involved primarily in the detection and regulation of signaling pathways triggered by β-oxidation metabolites, particularly hydroxycarboxylic acids. The plasma concentrations of these acids fluctuate significantly in response to metabolic states. Under conditions of elevated β-oxidation (the main method of fat oxidation and decomposition), such as fasting or diabetic ketoacidosis, levels of the intermediate 3-hydroxyoctanoic acid (3HO) rise sufficiently to activate HCAR3. Upon activation, HCAR3 signals through the G$\alpha_{i/o}$ protein, inhibiting adenylyl cyclase and thereby exerting an antilipolytic effect. Given its pivotal role in lipid metabolism, HCAR3 presents a promising therapeutic target for managing dyslipidemia and other metabolic disorders, including diabetes mellitus and obesity. While niacin activates HCAR3, it typically requires high concentrations (over 300 µM), making it difficult to precisely determine its EC_50_ value and to classify it as a full or partial agonist [15]. Although about a dozen compounds modulating HCAR3 have been reported, the repertoire remains limited compared to other GPCR targets. This notable ligand gap underscores the need for virtual screening to discover novel, selective compounds that can bind to and modulate HCAR3.

In this work, a computer-aided drug design (CADD) approach [16,17] was employed to discover potential candidates that can bind with HCAR3. CADD integrates various computational modeling and simulation techniques, including molecular docking [18], molecular dynamics (MD) simulations [19], and free energy calculations [20]. It allows us to virtually screen ligand databases such as ZINC [21] for potentially bioactive compounds. First, we identified the desired HCAR3 structure model for screening, followed by the absorption, distribution, metabolism, excretion, and toxicity (ADMET) prediction [22]. Computational analysis identified ARG251 and ARG111 as key residues for ligand recognition [23,24,25]. However, we prioritized ARG111 because it consistently formed stable interactions with all active compounds, is centrally positioned to anchor and orient ligands, and has been identified as a key residue in HCAR2 [25], providing a strong structural basis for ligand optimization in HCAR3. In the end, ligands with good docking scores and favorable interactions were then chosen for MD simulations to explore their binding stability. Finally, the associated binding affinities were computed using umbrella sampling [26], using the weighted histogram analysis method (WHAM) [27].

Given the limited availability of potent and selective HCAR3 modulators and the receptor’s critical role in lipid metabolism, this study was undertaken to identify novel ligands through an integrated CADD workflow, aiming to provide a rational foundation for developing targeted therapies against dyslipidemia and related metabolic disorders.

## 2. Results and Discussion

### 2.1. Cross-Docking Analysis and Homology Modeling of HCAR3

**Figure 1:**
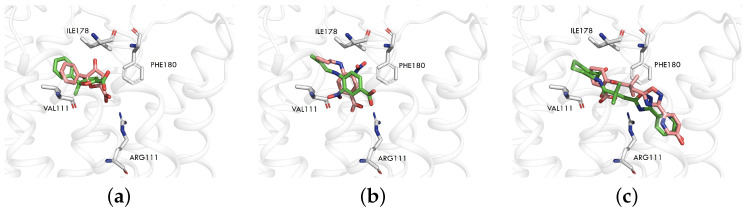
Comparison of ligand docking poses in *HCAR3_homology*. The three panels depict docking results for different ligands ((**a**) 8IHJ, (**b**) 8JEI, (**c**) *HCAR3_homology*). The receptor structure is derived from *HCAR3_homology* in all cases. The green stick representation shows the ligand docking pose, while the pink representation corresponds to the ligand conformation from the PDB structure.

To determine the optimal structure for virtual screening, we considered two HCAR3 cryo-EM structures (PDB codes: 8IHJ and 8JEI) and one HCAR3 homology model constructed based on HCAR2, which shares 95% sequence identity with HCAR3. For this homology model, we performed cross-docking using five HCAR2–ligand complex structures (PDB codes: 7XK2, 8IHB, 8IHF, 8IHH, and 8IHI) to select the best structural template. Each ligand was docked into the receptors using Smina, and the top-ranking docking pose was compared to the crystal ligand pose. The corresponding RMSD values are reported in Table 1. The diagonal values represent the RMSD between the docking pose and the ligand pose from the corresponding PDB structure. In our analysis, the RMSD values along the diagonal are very small, generally smaller than 1.5 Å, suggesting that Smina’s parameters for docking are acceptable. However, large RMSD values (up to 9.53 Å) were also observed when docking MK6892 (the experimental ligand of 7XK2 and 8IHF) to receptors from the 8IHB, 8IHH, and 8IHI structures. This can be attributed to the larger size of MK6892 compared to the ligands in these structures. Consequently, MK6892 fails to fit into the smaller binding pocket of these three receptors (Figure S2). Overall, the receptor with PDB code 7XK2 yielded the lowest average RMSD across a diverse range of ligands (Figure 1), indicating that it is the best template for docking among the five structures investigated. We then selected HCAR2 (7XK2) as the template to build the homology model for HCAR3, which was labeled as *HCAR3_homology*.

Similarly, we conducted a cross-docking for two HCAR3 structures (PDB codes: 8IHJ [25] and 8JEI [25]) and the homology model (*HCAR3_homology*). Again, ligands in these two PDB structures are much smaller than those of MK6892; hence, the two structures cannot accommodate MK6892, as reflected in the RMSD values shown in Table 1. Given that some reported active compounds for HCAR3 are relatively large, we eventually selected the homology model (*HCAR3_homology*) to accommodate such compounds in the virtual screening.

### 2.2. Retrospective Docking

**Figure 2:**
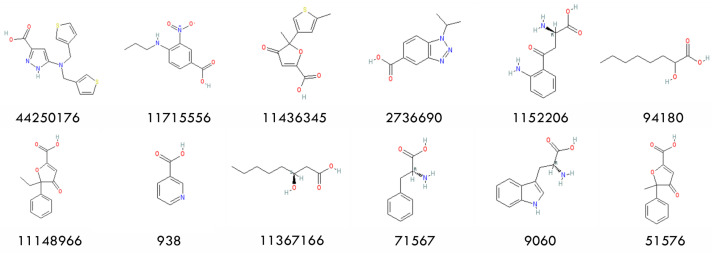
Chemical structures of the 12 actives and their PubChem CIDs.

**Figure 3:**
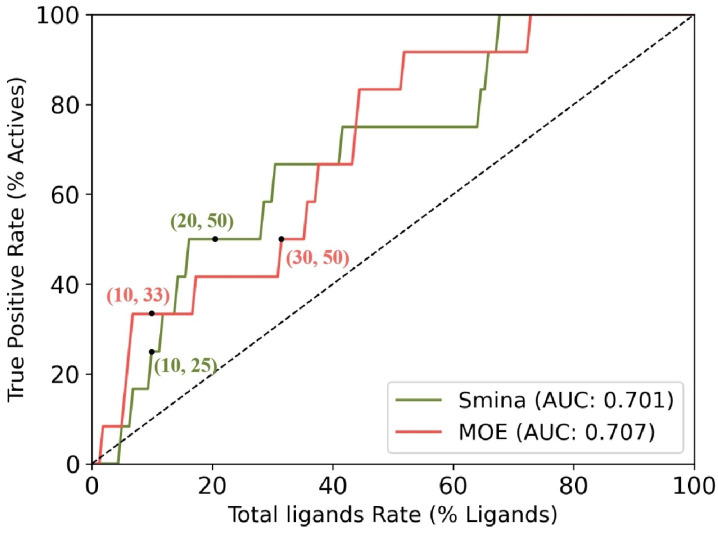
ROC curves from the retrospective docking. Both Smina (release compiled on 15 October 2019) and MOE (version 2020.09) software were tested. Here the true positive rate is plotted against the total ligand rate (percentage of ligands screened). The diagonal dashed line represents a random classification model (AUC = 0.5), serving as a baseline for performance comparison.

Upon observing the ligand–receptor interaction in the HCAR2 structure (7XK2), ARG111 was identified as an important residue for substrate recognition. Additionally, all 12 active compounds carry a carboxylate group (Figure 2). These two facts suggest that a salt bridge formed by the ARG111 carboxylate group is crucial and should be kept. Therefore, we restricted the screening to ligands containing carboxylate groups. Retrospective docking was conducted using a dataset of 12 active compounds and 150 decoys, all containing carboxylate groups. Both Smina and MOE were performed to evaluate their performance. Similar AUCs were found; see Figure 3 for ROC curves and AUCs. MOE demonstrated a better early enrichment, recovering 33% of active compounds within the top 10% of ranked ligands, whereas Smina recovered only 25%. However, Smina recovered 50% of actives within the top 20% ranked ligands, compared to MOE, which required the top 30%. Based on this performance, an efficient screening strategy would be a rapid initial screening with Smina, followed by a more refined screening using MOE.

### 2.3. Virtual Screening and ADMET Analysis

**Figure 4:**
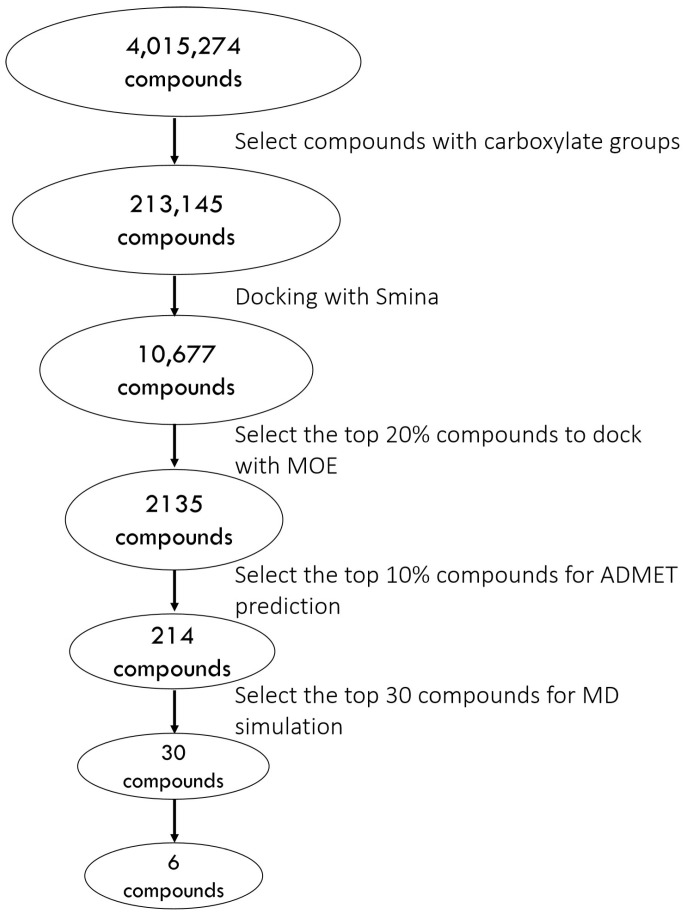
Flowchart of the virtual screening process.

**Figure 5:**
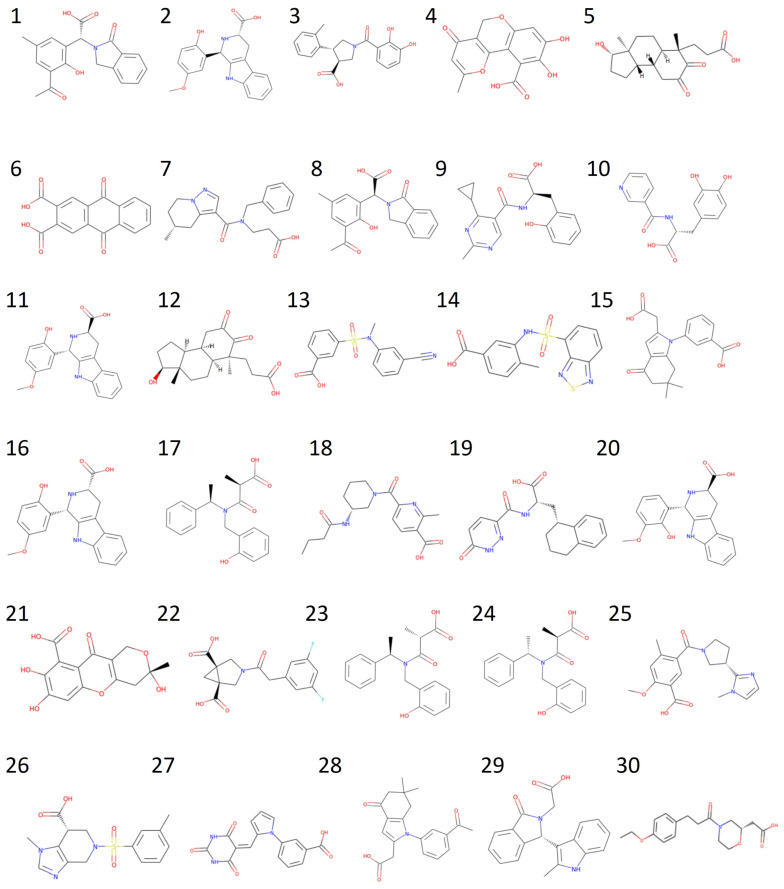
Chemical structures of the 30 compounds from virtual screening.

The results of the retrospective docking motivate us to build the following protocol. First, we screen the SMILES (simplified molecular input line entry system) from the ZINC database to filter for ligands with carboxylate groups. Details of the prospective docking using the 1D lead-like in-stock dataset (4,015,274 small compounds) are depicted in Figure 4. Upon screening for SMILES containing carboxylate groups, 213,145 compounds were left for docking, when using Smina and then MOE. After selecting the top 10% of compounds from the MOE docking results, we used ADMETlab 2.0 for ADMET screening. First, all candidates passed Lipinski’s Rule of Five, confirming drug-like properties. Next, 127 compounds with a logP value between 0 and 3 were retained, followed by 106 compounds with TPSA between 0 and 140. Among these, 93 compounds with a predicted probability of having a P-gp substrate below 0.5 successfully passed the PAINS filter, and 85 compounds with an Ames toxicity probability under 0.5 were further selected. Finally, the top 30 compounds based on docking scores were chosen for MD simulations. Table 2 presents the docking scores and ADMET predictions for the top 30 compounds, while their chemical structures can be found in Figure 5.

### 2.4. Molecular Dynamics Simulations of HCAR3–Ligand Complexes

To assess the stability and dynamic behavior of the HCAR3–ligand complexes, MD simulations were performed for the top 30 compounds. For each complex, three independent 100 ns simulations were conducted for analysis. The initial structure of a complex was taken from the corresponding ligand’s docking pose, where the ARG111–carboxylate interaction was kept (Figure S3).

**Figure 6:**
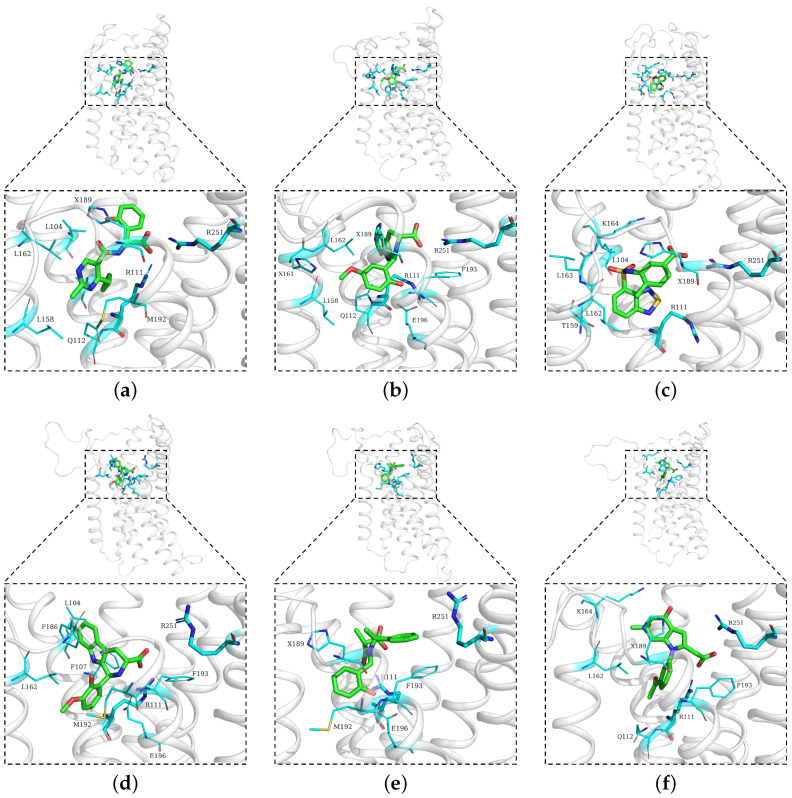
Representative structures of the main clusters for six compounds ((**a**) Compound 9, (**b**) Compound 11, (**c**) Compound 14, (**d**) Compound 20, (**e**) Compound 24, and (**f**) Compound 28). The overall protein is shown in white cartoon representation, with zoomed-in views highlighting the ligand-binding pocket. Ligands are shown in green, while residues within 3 Å of the ligands are displayed as cyan sticks. The key residues ARG111 and ARG251, which are the focus of this study, are shown as thicker cyan sticks for emphasis. The residue labeled with “X” represents $N_{\delta 1}$-protonated histidine.

Upon completing the simulations, the trajectories of the same complex were then appended together for analysis. In particular, the representative complex conformation was sought via cluster analysis, using an RMSD cutoff of 0.2 nm. As the major cluster was the group with the largest number of structurally similar neighbors, the cluster centroid is consequently the representative conformation, i.e., the major binding mode sampled during the simulations. Notably, only six compounds demonstrated a main cluster population exceeding 30%. They showed a stable major binding mode; some of these modes revealed an interaction of carboxylate with ARG251, see, e.g., Compound 9, Compound 11, and Compound 14 in Figure 6.

**Figure 7:**
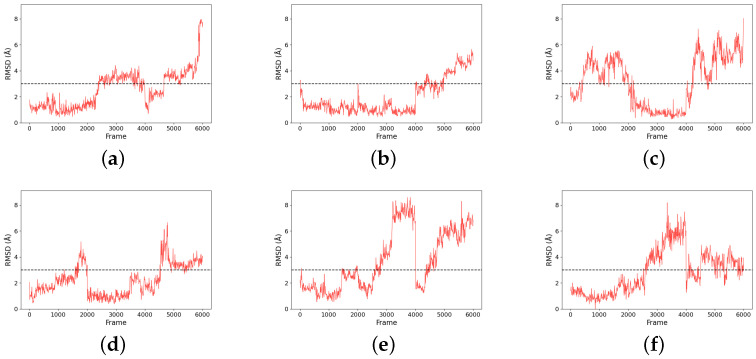
RMSD plots for six compounds ((**a**) Compound 9, (**b**) Compound 11, (**c**) Compound 14, (**d**) Compound 20, (**e**) Compound 24, and (**f**) Compound 28) compared to their representative structures (centroid of the major cluster) over the trajectories. Notably, frames 1–2000, 2001–4000, and 4001–6000 are trajectories from three independent simulations.

**Figure 8:**
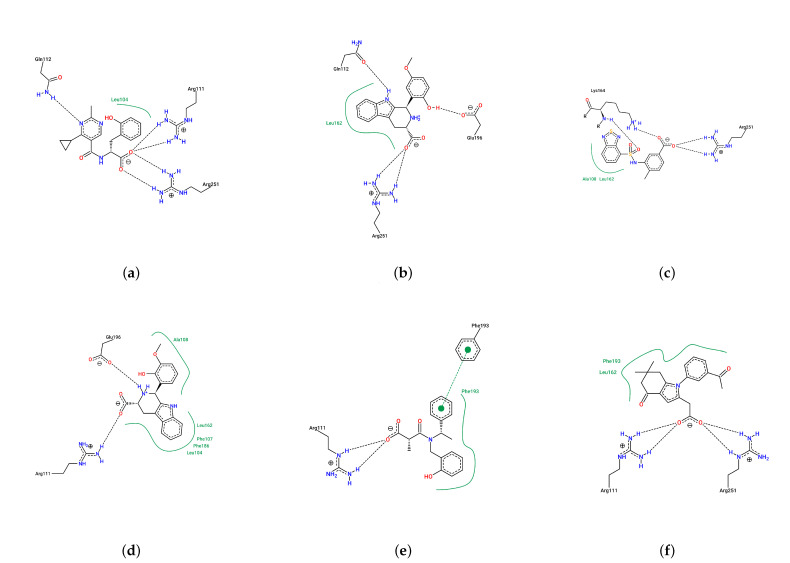
Two-dimensional representation of the intermolecular interactions between the selected compounds and HCAR3 in the simulated complexes ((**a**) Compound 9, (**b**) Compound 11, (**c**) Compound 14, (**d**) Compound 20, (**e**) Compound 24, and (**f**) Compound 28). Hydrogen bonds are shown as dashed lines. Green lines represent hydrophobic or π–π stacking interactions between aromatic rings.

The size of the major cluster reflects the stability of the binding mode discovered. In terms of the total simulation length, the major cluster represents 43.69% of Compound 9 simulations, 64.98% of Compound 11 simulations, 39.03% of Compound 14 simulations, 52.21% of Compound 20 simulations, 34.52% of Compound 24 simulations, and 41.83% of Compound 28 simulations. To better visualize the stability of the binding, all protein frames were aligned to the representative conformation; then, we calculated the ligand RMSD over the simulations, with respect to the major binding mode. An RMSD threshold of 3 Å was established as the criterion for acceptable protein structural stability throughout the simulations. As shown in Figure 7, most ligands exhibited stable RMSD values around 3 Å. Consequently, umbrella sampling was performed solely for these six compounds, with RMSD stability and the main cluster proportion (>30%) serving as the objective selection criteria. In particular, Compound 11 (panel b) has about 2/3 of frames with an RMSD below 2 Å. In contrast, Compound 24 has very few frames with a low RMSD. That means the binding mode found in Compound 24 simulations is not stable and could very well be incorrect. Such a result suggests that the initial docking pose may be incorrect, which could be a consequence of using a large binding pocket, within which two arginines (ARG111 and ARG251) could compete for carboxylate recognition. To further characterize the binding modes of the compounds, two-dimensional interaction diagrams were generated using PoseView [28]. In addition to the previously described interactions with arginine residues, the simulated complexes revealed diverse binding patterns involving multiple residues of HCAR3 (Figure 8). Compound 9 formed hydrogen bonds with GLN112 and hydrophobic contacts with LEU104. Compound 11 engaged GLN112 and GLU196 through hydrogen bonding, with LEU162 providing consistent hydrophobic contacts. Compound 14 was stabilized by hydrophobic interactions with ALA108 and LEU162, as well as hydrogen bonding with LYS164. Compound 20 exhibited the most extensive interaction network, forming hydrogen bonds with GLN196, complemented by hydrophobic contacts with LEU104, LEU162, PHE107, and PHE186. Compound 24 was further stabilized by π–π stacking with PHE193, while Compound 28 was stabilized by hydrophobic interactions with LEU162 and PHE193.

### 2.5. Free Energy Calculation via Umbrella Sampling

To quantify the binding strengths of the selected ligands, umbrella sampling was performed using GROMACS. The initial conformation of the protein–ligand complex was taken from the centroid of the major cluster of the MD simulations. The ligand was then pulled out from the protein pocket, and the PMF along this path was calculated according to WHAM. Details of the parameters can be found in the Methods section.

**Figure 9:**
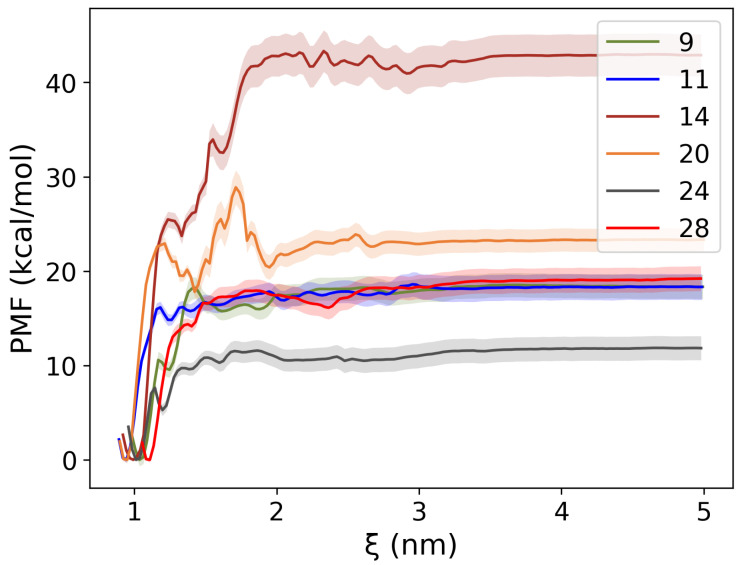
PMF profiles with bootstrap-derived error bars for six compounds. The potential well is the free energy difference between the ligand-bound state and the ligand dissociation limit, viz., the binding affinities. All compounds have negative binding affinities.

As depicted in Figure 9, all PMF free energy profiles exhibit a sharp increase at short distances (ξ ~1 nm), indicating that the ligand–protein interaction is strong within the binding pocket. Beyond this point, a plateau was observed as the ligand can be considered completely dissociated from the receptor under this inter-molecular distance. These PMF results provide crucial insights into the energetic landscape of ligand dissociation. For instance, the potential well depth is the free energy difference between the dissociation limit (ξ>3.5 nm) and the initial bound state, viz., the binding affinity of the ligand. Therefore, a quantitative comparison of ligand binding strength is possible. As shown in the panel, Compound 14 demonstrated the strongest binding among the six compounds (−40.23±2.23 kcal/mol), whereas Compound 24 has the weakest binding (−7.52±1.27 kcal/mol). Compound 9, Compound 11, and Compound 28 have similar binding affinities (−15.79±1.30, −15.82±1.42, and −16.66±1.32 kcal/mol, respectively), while Compound 20 has a binding affinity of −20.62±1.22 kcal/mol. Interestingly, for compound 9,11,14,28, the carboxylate interaction with ARG251 is present in the sampling. This further stresses the relevance of ARG251 in substrate recognition. We note that the calculated binding affinities may exceed the typical range for the standard binding free energy of small neutral ligands (approximately –5 to –15 kcal/mol). This is because the computed values represent the free energy required to dissociate a charged ligand from a counter-charged binding pocket, rather than the standard binding free energy. To avoid confusion, we use the term “binding affinity” instead of “binding free energy.”

## 3. Materials and Methods

### 3.1. Receptor Structure Comparison

Because rigid docking (which uses a fixed receptor conformation) is employed, the results of virtual screening are highly dependent on the quality of the structure used. To identify the most suitable HCAR3 structure for virtual screening, we compared a HCAR3 homology model with two Cryo-EM structures of HCAR3 [25] (PDB codes: 8IHJ and 8JEI) from the Protein Data Bank [29]. The HCAR3 homology model, denoted as *HCAR3_homology*, was constructed using MODELLER [30], using the HCAR2 structure 7XK2 as the template. HCAR2 belongs to the same subfamily as HCAR3, with a sequence similarity of 95%. Among the generated models, the one with the lowest molpdf score was selected as the final structure. The molpdf is MODELLER’s scoring function, with lower values indicating a better agreement with the template. We also performed structural alignments of the homology model with two experimentally determined HCAR3 structures (Figure S1). The RMSD between the homology model and 8IHJ was 1.316 Å, while between the homology model and 8JEI, it was 1.358 Å, indicating high structural similarity. Among the five Cryo-EM structures of HCAR2 (7XK2 [31], 8IHB [25], 8IHF [25], 8IHH [25], and 8IHI [25]), 7XK2 was chosen based on cross-docking results [32]. Cross-docking involves comparing ligand docking poses with experimentally resolved ligand poses across multiple protein–ligand complexes. The protein conformation that can accommodate the most ligands with the smallest root-mean-square-deviation (RMSD) is then selected for virtual screening. In this case, 7XK2 was selected as the template for homology modeling because its conformation can also accommodate larger active compounds. This approach allows for the discovery of both large and small molecules that might otherwise be excluded by more restrictive pocket geometries. All structural figures were generated using PyMOL [33] version 3.1.0.

### 3.2. Virtual Screening

The virtual screening of drug candidates from the ZINC database [21] was conducted via docking [34,35], which can rapidly estimate the binding pose of a compound in complex with the target protein, as well as the associated binding affinity. The virtual screening is composed of two stages—a retrospective docking and a prospective docking. The former aims to construct a validated docking protocol, which will then be applied to the latter for screening. Given that all known active compounds in the literature contain a carboxylate group, as well as ARG111 being a key residue for substrate recognition in the homologous protein HCAR2, we tested a protocol targeting only ligands with carboxylate groups. To evaluate the performance of this protocol, the dataset was compiled using the 12 known HCAR3 agonists (actives) from the IUPHAR/BPS Guide to PHARMACOLOGY [36] and 150 decoys, which were deliberately filtered from the DUD-E (A Database of Useful Decoys: Enhanced) web server [37] to yield decoys with carboxylate groups. Decoys are inactive ligands with similar physicochemical properties to the active compounds; however, their topologies are so different from actives that they should not bind with the target [37,38]. As for the performance of a docking protocol, it is assessed from its receiver operating characteristic (ROC) curves, together with the area under the curve (AUC). Larger AUC values, as well as those that demonstrate an early enrichment, are considered to be a better protocol.

Compounds were initially docked using Smina [39], which is a fork of AutoDock Vina [40] that allows users to easily implement their own scoring functions, although the default scoring function was employed in our screening. The docking boxes were automatically defined around the ligand with a 4 Å buffer. The exhaustiveness was set to 16, and the maximum number of binding modes generated was 9. The ligands were then further piped through Molecular Operating Environment (MOE) [41] for the second round of docking, using the ASE scoring function. This physically based scoring function was selected for its performance against active compounds, as it enables the accurate estimation of solvation energies, provides balanced scoring by integrating both solvation and interaction terms, and has been trained and validated on experimental data to produce predictions consistent with real binding trends. The binding pocket was identified using MOE’s Site Finder, considering both its size and position. Through a consensus approach, we can quickly remove ligands that are too big to fit into the binding pocket via Smina, while keeping good candidates among the top-ranked ligands identified by MOE. After completing retrospective docking, the validated protocol was applied prospectively to identify potential active compounds from the one-dimensional, lead-like, in-stock subset of the ZINC20 database.

### 3.3. ADMET Evaluation

To identify drug-like candidates with favorable pharmacokinetic profiles, we subjected compounds that passed the virtual screening filters to ADMET prediction. ADMET, which stands for absorption, distribution, metabolism, excretion, and toxicity, encompasses key properties for assessing the pharmacokinetic and safety profiles of potential drug candidates. While our docking protocols evaluated potential target engagement, ADMET analysis determined whether the compounds possessed the drug-like properties necessary for further development. This computational approach helps eliminate molecules with unfavorable pharmacokinetic properties early in the drug discovery pipeline, providing valuable feedback for lead optimization and reducing the risks of later-stage attrition. For our ADMET assessment, we used ADMETlab 2.0 [42], which is a comprehensive platform for predicting the druggability of small molecules. Six key parameters were evaluated—Lipinski’s rule of five, octanol–water partition coefficient (logP), topological polar surface area (TPSA), P-glycoprotein (P-gp) substrate status, pan-assay interference compounds (PAINS), and the Ames test—providing a comprehensive evaluation of candidate molecules. By setting these parameters, we aimed to filter out non-druggable molecules early in the screening process and focus on candidates with promising pharmacokinetic and safety profiles, thereby streamlining lead optimization and increasing the likelihood of successful drug development.

### 3.4. Molecular Dynamics Simulations

Molecular dynamics simulations were performed using GROMACS [43] to evaluate the stability and dynamic behavior of protein–ligand complexes. The initial binding models were derived directly from the docking results, with protonation states being determined according to propKa [44] at a physiological pH of 7.0. To simulate the native membrane environment, we constructed and parametrized a cell membrane using ChARMM-GUI [45,46,47], with a membrane composed of cholesterol and DOPC at a 1:9 ratio. The system was then solvated in a 0.15 M NaCl solution within a rectangular box of dimensions 81.4 × 81.4 × 100.9 Å^3^. CHARMM36m [48] and CGenFF [49] force fields were employed for the protein/membrane and ligands, respectively. The simulation protocol began with a 5000-step energy minimization using the steepest descent method to remove high-energy contacts. This was followed by a two-phase equilibration—1.5 ns in the NVT ensemble and 3.75 ns in the NPT ensemble at 303.15 K and 1 bar pressure—where the position restraints on the system and lipids are gradually lifted. In the NPT phase, restraint force constants were reduced stepwise from 4000 to 2000, 1000, 500, 200, 50, and, finally, 0 $kJ//nm^{2}$. All restraints were removed before the production simulations. The resulting trajectories were combined and analyzed using the Gromos clustering algorithm [50] to identify representative conformations.

### 3.5. Free Energy Calculations

The binding affinity of the protein–ligand complexes was also calculated using GROMACS. The initial structures for these calculations were taken from the central conformation of the main cluster obtained from our MD simulations. The binding affinity ($\Delta G_{\mathrm{bind}}$) was derived from the potential of mean force (PMF), i.e., the free energy profile along the reaction path. For our purpose, the reaction coordinate was chosen to be the distance between the center of mass (COM) of the ligand and the COM of the protein. Steered MD simulations were utilized to generate the ligand unbinding path for the PMF calculation via umbrella sampling (US) [26]. The ligand pulling direction in the steered MD simulations was defined along the positive z-axis, as the HCAR3 binding pocket opens along this direction. To sample the conformations along the reaction path properly, the sampling was divided into several windows; in each window, harmonic biasing potential was applied, as follows: $V_{\mathrm{bias}}\left(x\right)=\frac{1}{2}k{\left(x-x_{0}\right)}^{2}$, where *x* represents the reaction coordinate, $x_{0}$ denotes the center of the potential, and *k* is the force constant. The force constant was set to 1000 $kJ//nm^{2}$; however, in a few cases, it was increased to 25,000 $kJ//nm^{2}$ to ensure the sampling occurred at the desired coordinate value. Each ligand was sampled using approximately 100 windows. Finally, the free energy profile was reconstructed using the weighted histogram analysis method (WHAM) [27]. To estimate the uncertainty of the PMF profiles obtained from umbrella sampling, we performed bootstrapping with 100 resampling iterations. The standard error was calculated at each reaction coordinate to generate the corresponding error bars.

## 4. Conclusions

Owing to its pivotal role in lipid metabolism, we conducted a virtual screening study targeting HCAR3 to identify potential drug candidates for dyslipidemia. Computational tools, including homology modeling, molecular docking, molecular dynamics simulations, and umbrella sampling, were employed for this purpose. In this study, we enhanced the performance of retrospective docking for the potential drug target HCAR3 by prioritizing compounds containing carboxylate groups and utilizing two different docking programs. This approach yielded a promising AUC value of 0.707. This performance is attributed to the key interaction between HCAR3 and the active compounds via a salt bridge involving ARG111. Following the successful establishment of an optimized docking protocol, we performed virtual screening against the ZINC20 database to identify novel compounds that may bind to HCAR3. Subsequently, we applied ADMET filtering and selected the top 30 compounds for MD simulations. From these, six compounds were ultimately chosen for umbrella sampling to calculate binding free energies. In total, 4,015,274 compounds were screened, leading to the identification of six promising candidates. Additionally, our MD simulations and umbrella sampling revealed that ARG251 is an important residue that interacts with the carboxylate group. These findings lay the groundwork for further experimental validation and the development of HCAR3-targeted therapeutics for dyslipidemia.

## Figures and Tables

**Table 1:** RMSD (Å) of ligands, measured between the docking poses and the cryo-EM structures. The docking is performed using each ligand and each receptor from the experimentally resolved structures. The values along the diagonal of the table are self-docking results, while off-diagonal values represent cross-docking results.

	Receptor	7XK2	8IHB	8IHF	8IHH	8IHI	8IHJ	8JEI	*HCAR3_homology*
Ligand									
MK6892 (7XK2)		2.18	9.53	1.90	6.98	9.45			
GSK256073 (8IHB)		4.65	1.18	6.77	2.53	2.13			
MK6892 (8IHF)		2.05	9.39	1.13	6.94	9.14			
LUF6283 (8IHH)		1.85	1.41	4.68	1.24	1.82			
Acifran (8IHI)		3.11	1.48	4.73	1.78	1.20			
Acifran (8IHJ)							0.83	2.90	2.30
Compound 5c (8JEI)							3.75	1.86	3.78
MK6892 (*HCAR3_homology*)							6.57	9.83	2.82

**Table 2:** Molecular characterization of the top 30 compounds.

Compound	Docking Score	Lipinski Rule	Log P	TPSA	Pgp-Substrate	PAINS	AMES
1	−14.1521	Accepted	2.779	94.91	0.019	1	0.005
2	−13.6680	Accepted	0.174	94.58	0.003	1	0.025
3	−13.6651	Accepted	2.304	98.07	0.006	1	0.006
4	−13.6480	Accepted	1.464	117.2	0.059	1	0.251
5	−13.6066	Accepted	1.294	91.67	0.004	1	0.016
6	−13.5977	Accepted	2.127	108.74	0.001	1	0.045
7	−13.4622	Accepted	2.252	75.43	0.003	0	0.015
8	−13.4273	Accepted	2.779	94.91	0.019	1	0.005
9	−13.2929	Accepted	1.275	112.41	0.066	0	0.014
10	−13.1917	Accepted	0.341	119.75	0.003	1	0.015
11	−13.1674	Accepted	0.408	94.58	0.05	1	0.025
12	−13.1380	Accepted	1.294	91.67	0.004	1	0.016
13	−13.1344	Accepted	2.443	98.47	0	0	0.013
14	−12.9854	Accepted	0.695	112.74	0.001	0	0.03
15	−12.9844	Accepted	2.757	96.6	0.004	1	0.022
16	−12.9796	Accepted	0.408	94.58	0.05	1	0.025
17	−12.9611	Accepted	2.776	77.84	0.005	1	0.007
18	−12.9402	Accepted	0.913	99.6	0.035	0	0.004
19	−12.9013	Accepted	0.757	107.53	0.003	0	0.049
20	−12.8954	Accepted	0.356	94.58	0.117	1	0.01
21	−12.8953	Accepted	0.882	137.43	0.02	1	0.14
22	−12.8655	Accepted	0.299	94.91	0.018	0	0.089
23	−12.8398	Accepted	2.776	77.84	0.005	1	0.007
24	−12.8298	Accepted	2.96	77.84	0.114	1	0.008
25	−12.8096	Accepted	0.562	84.66	0.012	0	0.006
26	−12.8093	Accepted	0.278	92.5	0.011	0	0.015
27	−12.8089	Accepted	1.333	117.5	0.004	1	0.082
28	−12.7568	Accepted	2.556	76.37	0.002	1	0.16
29	−12.7276	Accepted	2.55	73.4	0.452	0	0.009
30	−12.6631	Accepted	1.01	76.07	0.009	0	0.077

*Docking scores reported here are taken from MOE. Lower values suggest stronger binding. All other values were retrieved from ADMETlab 2.0.*

## Data Availability

Data are contained within the article and Supplementary Materials.

## Supplementary Materials

The following supporting information can be downloaded at https://www.mdpi.com/article/10.3390/ph18091290/s1. Figure S1: Structural alignment of the HCAR3 homology model with experimentally determined HCAR3 structures. (A) Alignment of the HCAR3 homology model (cyan) with 8IHJ (green). (B) Alignment of the HCAR3 homology model (cyan) with 8JEI (pink). Figure S2: Comparison of MK6892 binding poses across different receptor structures. Each panel (A-J) shows receptor-ligand interactions, with receptors depicted as cartoons and colored by PDB ID: white (7XK2), magenta (8IHF), cyan (8IHB), yellow (8IHH), and orange (8IHI). Residues within 3 Å of the ligand are shown as sticks and colored to match their respective receptors. The green stick representation shows the ligand docking pose, while the pink representation corresponds to the ligand conformation from the PDB structure. The pink stick representations in panels A, C, E, G, and I are MK6892 derived from 7XK2, while those in panels B, D, F, H, and J are MK6892 derived from 8IHF. Figure S3: Docking poses of six selected compounds (A: Compound 9, B: Compound 11, C: Compound 14, D: Compound 20, E: Compound 24, F: Compound 28). The overall protein is shown in white cartoon representation, with zoomed-in views highlighting the ligand-binding pocket. Ligands are shown in green, while residues within 3 Å of the ligands are displayed in cyan sticks. The key residues ARG111 and ARG251, which are the focus of this study, are shown in thicker cyan sticks for emphasis. The residue labeled with “X” represents $N_{\delta 1}$-protonated histidine. Figure S4: Molecular dynamics simulation movies of six different compounds (A: Compound 9, B: Compound 11, C: Compound 14, D: Compound 20, E: Compound 24, F: Compound 28). Each panel corresponds to a movie showing the MD trajectory of a distinct compound. The receptor is displayed in white cartoon representation. ARG111 is shown as green sticks, ARG251 as purple sticks, and the ligand as blue sticks.
