# Supplementary material for: Structure Modeling and Virtual Screening with HCAR3 to Discover Potential Therapeutic Molecules
Source: Pharmaceuticals (Basel). 2025 Aug 28;18(9):1290. doi: 10.3390/ph18091290 (PMC12472694; doi:10.3390/ph18091290)
Supplement: Supplementary file 1 [file pharmaceuticals-18-01290-s001.zip › pharmaceuticals-3807605-supplementary.pdf]

Supplementary Figures

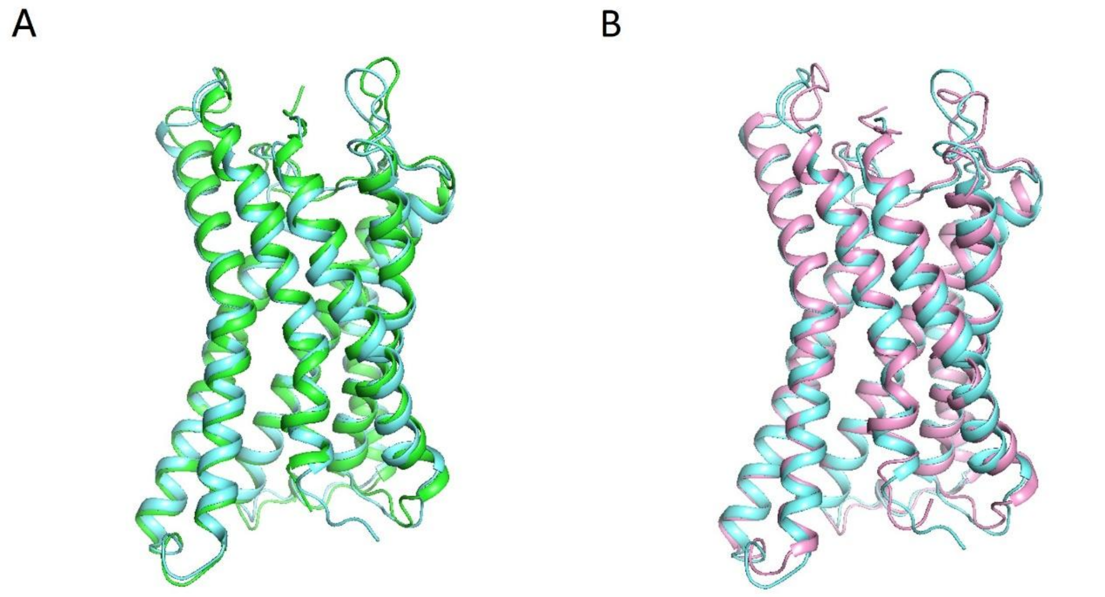

Figure S1: Structural alignment of the HCAR3 homology model with experimentally determined HCAR3 structures. (A) Alignment of the HCAR3 homology model (cyan) with 8IHJ (green). (B) Alignment of the HCAR3 homology model (cyan) with 8JEI (pink).

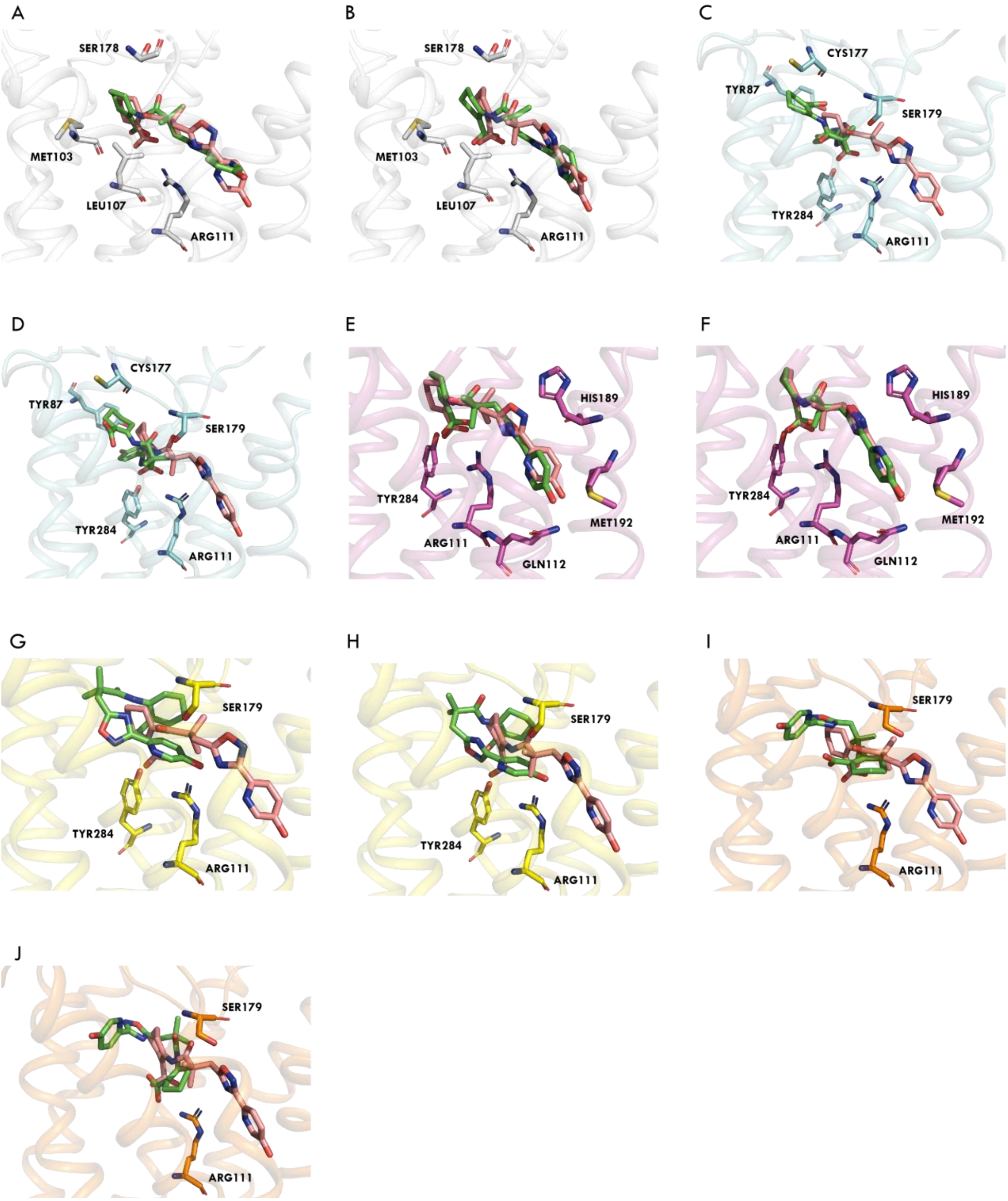

Figure S2: Comparison of MK6892 binding poses across different receptor structures. Each panel (A-J) shows receptor-ligand interactions, with receptors depicted as cartoons and colored by PDB ID: white (7XK2), magenta (8IHF), cyan (8IHB), yellow (8IHH), and orange (8IHI). Residues within 3 Å of the ligand are shown as sticks and colored to match their respective receptors. The green stick representation shows the ligand docking pose, while the pink representation corresponds to the ligand conformation from the PDB structure. The pink stick representations in panels A, C, E, G, and I are MK6892 derived from 7XK2, while those in panels B, D, F, H, and J are MK6892 derived from 8IHF.

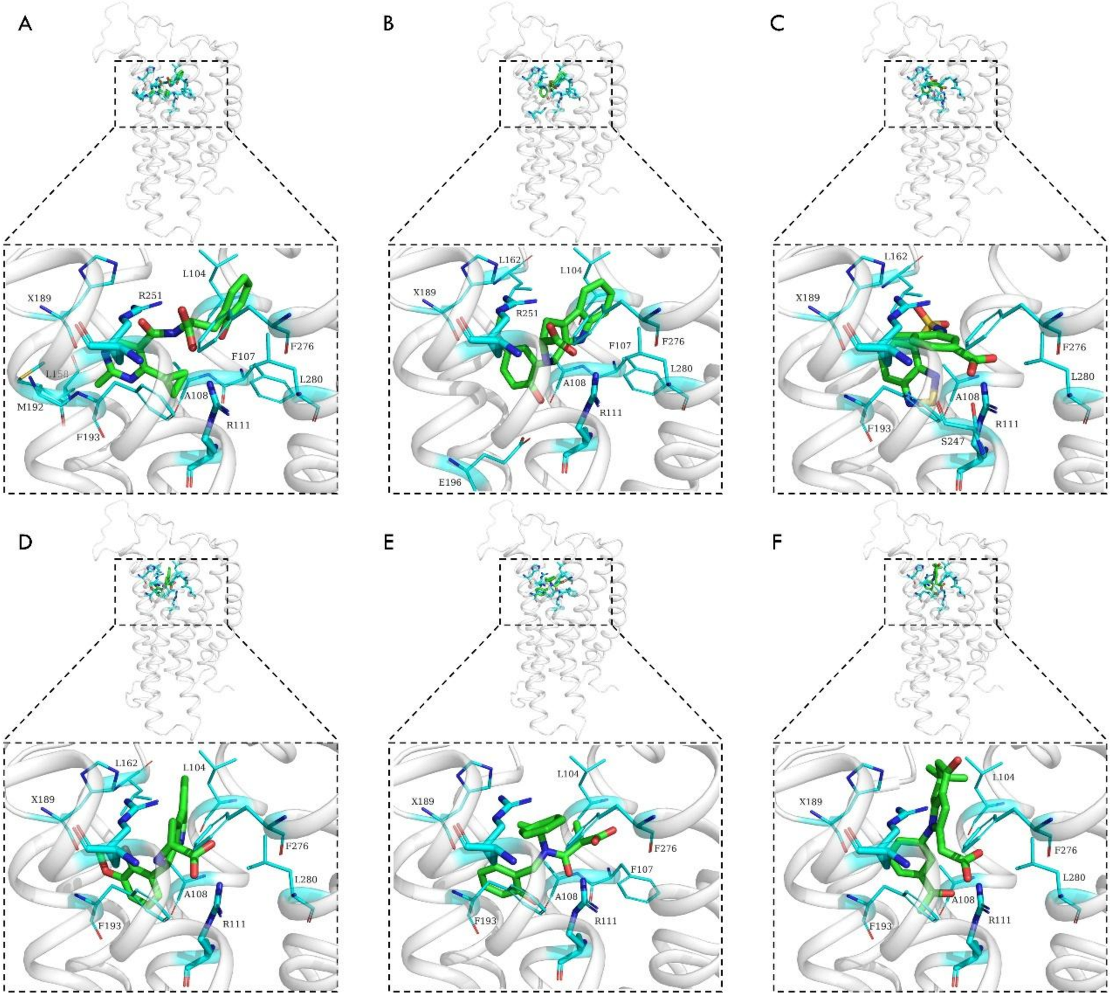

Figure S3: Docking poses of six selected compounds (A: Compound **9**, B: Compound **11**, C: Compound **14**, D: Compound **20**, E: Compound **24**, F: Compound **28**). The overall protein is shown in white cartoon representation, with zoomed-in views highlighting the ligand-binding pocket. Ligands are shown in green, while residues within 3 Å of the ligands are displayed in cyan sticks. The key residues ARG111 and ARG251, which are the focus of this study, are shown in thicker cyan sticks for emphasis. The residue labeled with "X" represents $N_{\delta 1}$ protonated histidine.

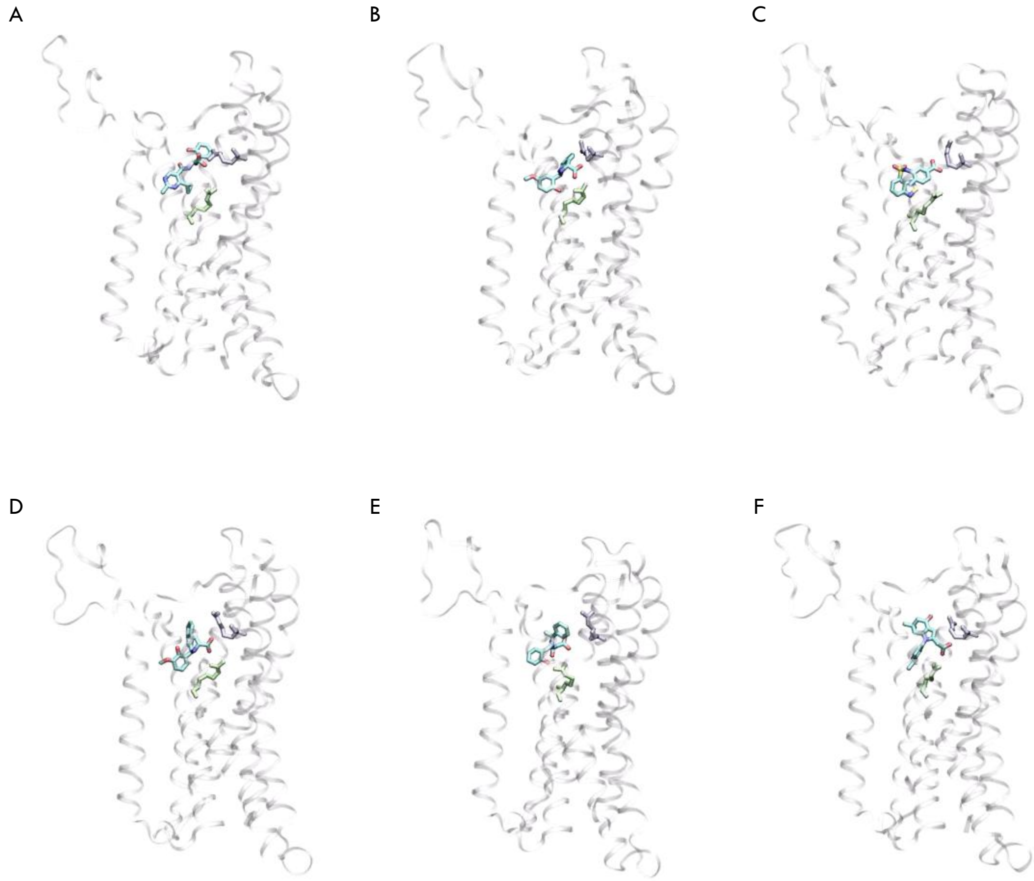

Figure S4: Molecular dynamics simulation movies of six different compounds (A: Compound **9**, B: Compound **11**, C: Compound **14**, D: Compound **20**, E: Compound **24**, F: Compound **28**). Each panel corresponds to a movie showing the MD trajectory of a distinct compound. The receptor is displayed in white cartoon representation. ARG111 is shown as green sticks, ARG251 as purple sticks, and the ligand as blue sticks.
